# Facilitators and barriers to the delivery of palliative care to patients with Parkinson’s disease: a qualitative study of the perceptions and experiences of stakeholders using the socio-ecological model

**DOI:** 10.1186/s12913-023-09203-2

**Published:** 2023-03-06

**Authors:** Yiping Chen, Ru Zhang, Yan Lou, Wei Li, Hui Yang

**Affiliations:** 1 School of nursing, Shanxi Medical University, Taiyuan City, Shanxi Province China; 2 Hangzhou Normal Unviersity, Hangzhou, Zhejiang Province China; 3 International Medical Department, Peking Union Medical College Hospital, Beijing, China; 4 Department of Neurology, First Hospital of Shanxi Medical University, No.56, Xinjian South Road, Yingze District, Taiyuan City, Shanxi Province China

**Keywords:** Parkinson’s disease, Palliative care, Facilitator, Barrier, Qualitative study

## Abstract

**Objective:**

Palliative care (PC) can improve the quality of life of Parkinson’s disease (PD) patients and their carers. However, the impact of PC services on patients with PD remains unclear. This research was conducted to identify the barriers and facilitators influencing PC services for patients with PD based on the Social Ecological Model (SEM) framework.

**Methods:**

This research was conducted through semi-structured interviews, employing SEM to organize themes and identify potential solutions across multiple levels.

**Results:**

A total of 29 interviewees (5 PD clinicians, 7 PD registered nurses, 8 patients, 5 caregivers, and 4 policy makers) completed the interviews. Facilitators and barriers were identified according to the levels of the SEM. Several facilitators were identified, i.e., (1) individual level: the critical needs among PD patients and their relatives and the desire for PC knowledge among health professionals; (2) interpersonal level: social support; (3) organizational level: the investments towards systematization of PC; and nurses are the bridge between patients and doctors; (4) community level: the convenience of community services; and hospital-community-family-based services; (5) culture and policy level: existing policy.

**Conclusion:**

The social-ecological model proposed in this study helps illuminate the complex and multilevel factors that may influence PC delivery to PD patients.

**Supplementary Information:**

The online version contains supplementary material available at 10.1186/s12913-023-09203-2.

## Background

Parkinson’s disease (PD) is a chronic neurodegenerative disorder currently incurable [1]. Recent findings demonstrate that PD is the fastest-growing neurological disease [2], with the prevalence of PD in China at 1.7% among adults over the age of 65 years. The number in China is expected to reach 5 million by 2030 compared to 1.99 million in 2005, almost half of the global number of patients with PD. Patients with PD generally experience diverse symptoms [3], including slow movement, body pain, anxiety, fatigue, sleeping problems, cognitive deficits, and dementia. Due to its slow progression, PD has not been considered a terminal disease in the early stages. However, as PD progresses to later stages, patient’s physical and psychosocial needs grow, increasing the burden on their carers. Although studies have shown that PD does not affect patient’s life expectancy [4, 5], many patients with PD die from complications of the disease, such as fall-related injuries, dementia, pneumonia, or infections, which indirectly contribute to increased mortality [6–8]. Therefore, paying attention to PD symptoms and improving the quality of life of patients with advanced PD can help reduce the premature death rate of patients.

The World Health Organization (WHO) states, “Palliative care (PC) is an approach (through the prevention and relief of suffering, early identification, impeccable assessment, and treatment) to improve the quality of life of patients and their families, those who are facing the pain and other physical, psychological and spiritual problems associated with a life-threatening illness [9].” There are about 40 million people globally who require palliative care and 78% live in low-and-middle-income countries where palliative care is in primitive stage [10]. For instance, on the basis of various dimensions of palliative care, Nepal is classified as ‘Category 3a’ with isolated palliative care provision [11]. This means that the development of palliative care services is still patchy in scope and not well supported. According to the WHO, PC is holistic care for patients and their families, including physical, psychological, social, and spiritual aspects. Long provided primarily for cancer patients, the incorporation of PC in managing various chronic diseases has subsequently been extensively studied, including dementia [12], chronic kidney disease [13], chronic obstructive pulmonary disease [14], and heart failure [15]. However, because the clinical trajectory of chronic disease is unpredictable compared to cancer, coupled with the fact that the focus in most countries remains on caring for patients with advanced cancer, the development of PC in chronic relatively slowly [16]. Research into the application of PC in PD management is currently growing [17], with a focus on its clinical benefits for PD patients. Recently, a large multi-center randomized controlled trial [18] was conducted to confirm the effectiveness of PC in patients with PD. The study revealed that PC recipients among PD patients experience a better life and treatment quality (i.e., motor and non-motor symptoms management, goals of care, anticipatory guidance, complex emotions management, and caregiver support) than the non-recipient.

Despite the advantages of PC in PD treatments, its application is still developing in many countries, including China [19]. Current research has also primarily focused on developed countries [20], neglecting the patients in developing countries, leading to dire prognoses. Therefore, this study was conducted to identify the barriers and facilitators influencing the provision of PC services to PD patients based on the social-ecological theoretical framework to improve the quality of the services. Theories derived from the findings made in this study will be helpful for the development of related policies and programs. At present, the practice of palliative care for patients with Parkinson’s disease in China [21] is in a preliminary exploratory stage and faces many problems, remaining in the management of symptoms alone, with no standardized implementation criteria or expert consensus. This study focused on Chinese healthcare professionals, patients, caregivers, and policymakers, which may be informative for other countries planning to develop PC services for PD patients.

## Methods

### Design

An explorative qualitative design was adopted in this study. The research was carried out in a province in China’s central area with poorer economic conditions and medical environment than that of the eastern region.

### Participants

The recruitment and interviews were conducted between September 2021 and May 2022 to obtain qualitative data. Recruitment posters were published by two researchers in six hospitals in different locations. The recruitment framework for the study was determined based on the socio-ecological model (SEM), where SEM divides the systems that influence an individual’s behavior from the inside out into a microsystem environment, a mesosystem environment and a macrosystem environment [22]. The microsystem refers to the healthcare professionals in the setting who specialize in providing palliative care services; the meso systems refer to the people in the setting who provide other services to individuals and are not specialized in providing palliative services, including families, occupational groups or other social groups; and the macrosystem refers to those who develop and implement high level guidelines and policies designed to support high quality palliative care(Supplementary files). Healthcare professionals recruited were asked to have at least three years of experience working with Parkinson’s and knowledge of palliative care, and people with Parkinson’s and caregivers were asked to have a desire to learn about or already know about palliative care. An invitation email was sent by the researcher to policy makers with experience of working in neurology and they were contacted when a response was received. To guarantee we had access to a variety of factors influencing stakeholders’ perceptions of palliative care, we employed maximum variance sampling. Our sample involves participants with different socio-demographic backgrounds, different ages and levels of education and different job titles. At first, a total of 60 (12 PD clinicians, 14 PD registered nurses, 16 patients, 10 caregivers, and 8 policy makers were recruited and invited. However, a total of 20 individuals were ultimately excluded, including those who were transferred to other institutions due to comorbidities, had died, could not be contacted, or had refused participation(n = 6) due to time constraints or discomfort. The final sample comprised five PD clinicians, seven registered PD nurses, eight patients, five caregivers, and four policymakers.

### Interview guides

The interview guides were developed using general, open-ended, and non-leading questions to determine the level of understanding among the participants regarding PC services in the context of PD, including the practical experiences in the area, the additional resources needed, the barriers and facilitators associated with the implementation of PC for PD patients, and the contexts in which PC for PD patients has been implemented (Supplementary files). For each type of interviewee(healthcare professional, patient, caregiver and policy maker), 1 or 2 interviewees was pre-selected for the pilot interview and the final interview guideline was formed, and the interviewees for the pilot interview didn’t included in the final interview (they are not in the formal recruitment process). The interviewer encouraged the investigation of responses using a blend of traditional interview approaches (i.e., probing questions, seeking clarification, confirming answers if required, and presenting reflections). In addition, the authors reviewed and examined new themes from the data collected regularly. Some of the questions were pre-designed for all medical practitioners, while some were spontaneously developed during one of the interviews and were included in the subsequent interviews.

### Data collection

The interviews were conducted separately by two researchers, both registered nurses, licensed to practice, with extensive experience in the chronic management of PD and not previously known to the interviewees. Most interviews were conducted in the neurology department and took place on a one-to-one basis so that participants could obtain meaningful information. A small number of interviews were conducted in other settings, such as a café. All participants signed a written consent form prior to the interview. Interviews lasted between 60 and 90 min.

### Data analysis

All semi-structured interviews were recorded on audiotape. Recordings were transcribed verbatim and the interviewees were subsequently asked to check their own interview text and were analysed by two researchers using thematic analysis, with an inductive approach and a coding strategy [23]. To increase the credibility of the analysis, the transcripts were de-identified and read and coded independently by the two research members. Firstly, a thematic framework was developed based on the purpose of the study and the interview outline, based on which the interviews were categorized and summarized, resulting in a more refined thematic framework containing several themes and corresponding level 1 indicators, and the coding was compared and discussed to form the coding sheets. To achieve inter-coder reliability, we undertook ongoing iterative consensus building and regular team meetings. If necessary, codes were discussed, added, updated, or merged. After coding the sample data profile, the coded thematic entries were collated and those with the same concept or similar meaning were expressed in a uniform manner, counting the frequency of occurrence of each code. No new themes emerged following the analysis of the 29 interviews, indicating that data saturation had been reached. Finally, theoretical coding was conducted using SEM, which highlights the interconnectedness of systemic processes with individual attitudes and perceptions [24]. Themes reflecting interviewees’ views and experiences regarding palliative care were labelled and categorized according to the theoretical framework. It was not until the final stage of data analysis that we applied SEM to our data to avoid preconceptions and biases in the initial transcript reading. NVivo software was used to organize and store the data and to manage the coding process.

## Results

### Participant characteristics

The participants included twelve healthcare professionals, eight patients, five caregivers, and four policymakers. The demographics of the participants in this study is presented in Table 1.

**Table 1:** Characteristics of the participant interviewed

	Healthcare professionals(n = 12)	Patients(n = 8)	Caregivers(n = 5)	Policy makers(n = 4)
Age, median(range)	33(26–45)	61(55–73)	60(53–67)	46(40–55)
Female, n(%)	8(68)	4(50)	5(100)	2(50)
Highest Education Completed, n(%)				
High school	0(0)	4(50)	4(80)	0(0)
College graduate	5(40)	4(50)	1(20)	1(25)
Master’s degree or higher	7(60)	0(0)		3(75)
Title, n(%)				
Associate Chief Physician	3(25)	-	-	-
Attending Physician	2(17)	-	-	-
Nurse Supervisor	5(42)	-	-	-
Nurse Practitioner	2(17)	-	-	-
Occupation, n(%)				
Retired	-	5(62)	2(40)	-
Employed	-	1(12)	0(0)	-
Unable to work	-	2(25)	3(60)	-
Perceived financial situation, n(%)				
Comfortable	-	3(38)	1(20)	-
Modestly comfortable	-	2(25)	2(40)	-
Tight	-	2(25)	2(40)	-
Poor	-	1(13)	0(0)	-

### Major themes

**Fig. 1:**
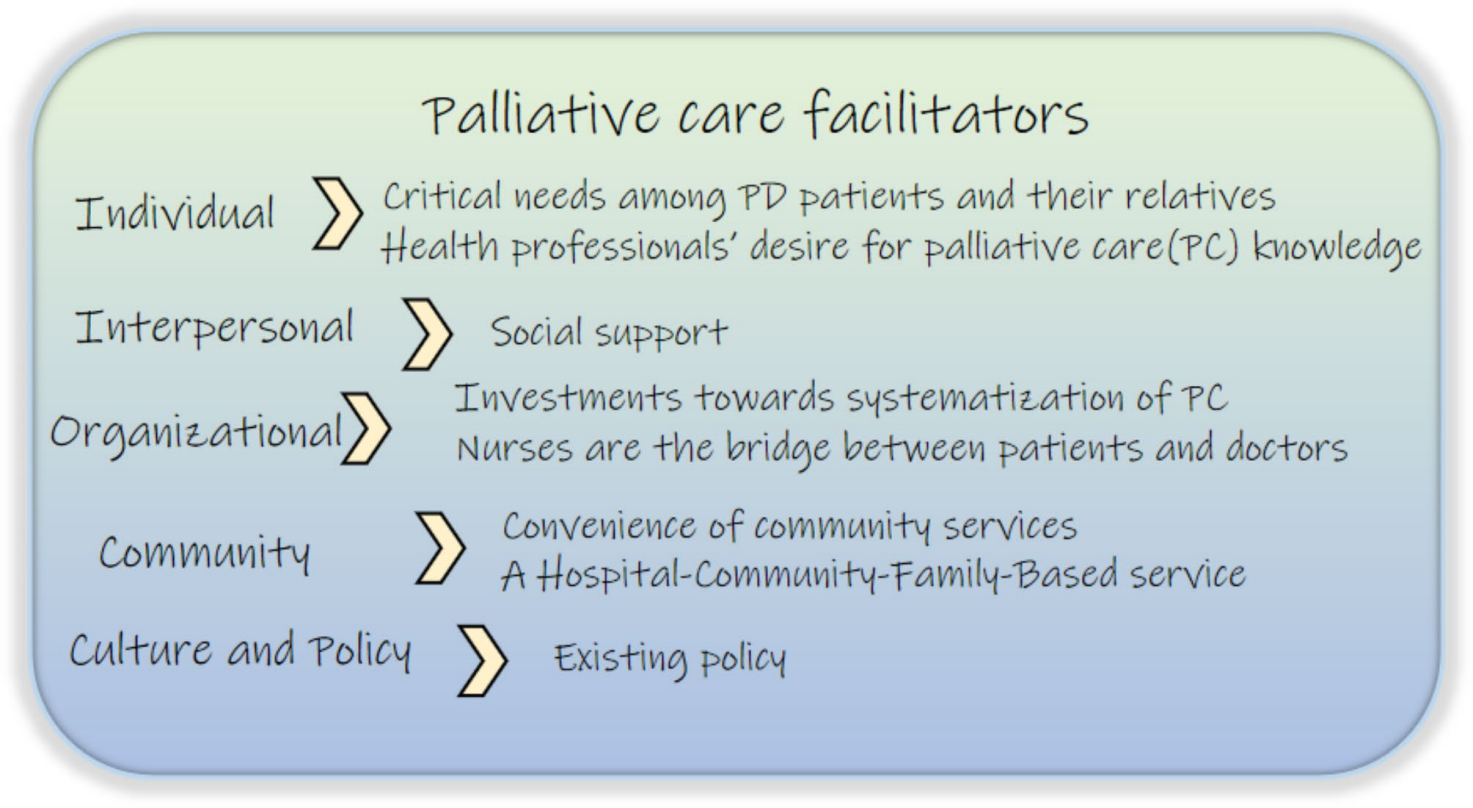
Palliative care facilitators for patients with Parkinson’s disease

**Fig. 2:**
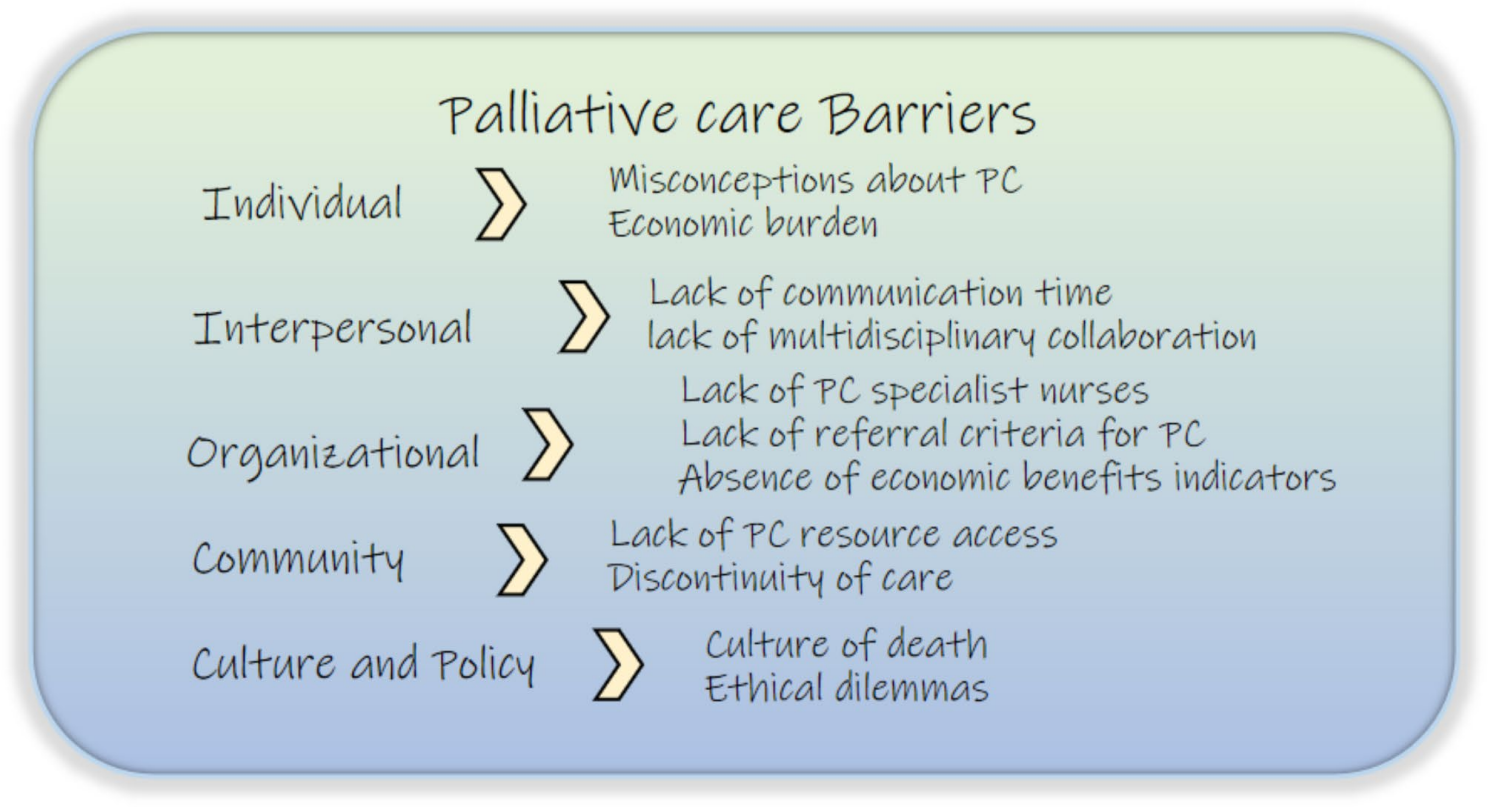
Palliative care barriers for patients with Parkinson’s disease

The codes and themes from the analysis were organized, and the social-ecological model was used to interpret the data (Figs. 1 and 2). Facilitators and barriers impacting PC delivery for patients with PD were identified at the individual, interpersonal, organizational, community, cultural and policy levels.

#### Individual level

At the individual level, two main themes facilitating PC were identified: the critical needs among PD patients and their relatives and the desire for PC knowledge among health professionals. However, Misconceptions about PC and economic burden were identified as barriers to PC delivery for patients with PD.

##### **Critical needs among PD patients and their relatives**

Patients with advanced PD typically experience multiple symptoms, including facial motor impairments such as muscle rigidity, tremor, bradykinesia, and ataxia. In addition, patients may experience psychiatric changes such as disturbances in consciousness, depression, hallucination, and delusion. Therefore, the patients often have a low quality of life.

“In general, patients in the advanced stages are largely bedridden, they are unable to care for themselves, their quality of life is poor, and they require our close care, they have problems with eating and drinking, they also have swallowing disorders, incontinence and may develop pressure sores.” [Healthcare professionals].

Most patients and family members have limited knowledge about PD, but a strong desire to learn. They have also reported feeling anxious and depressed in the early stages of the disease and having a lack of understanding about the prognosis of their condition. Consequently, these patients have constantly worried about their future, making them eager to communicate with their healthcare providers to understand their medical needs better. Most patients were also reportedly embarking on the internet searching to obtain information about PD and the lifestyle they should be expecting living with the disease. Family members have also expressed the critical need to learn about PD and the available treatments considering their long-term commitment to taking care of the patients.

“I will always communicate with my health care provider, for example, can I be cured of this disease, what is the best way for me to do this, how can I relieve this symptom of mine after treatment, what can I do to improve the quality of life with this disease …….” [Patient].

“Many patients and families have this strong desire to learn, and he has this condition in himself that can support him to learn things. We need to reach out to them about these things.” [Healthcare professional].

PC has been associated with improving the quality of life. PC is designed to provide professional disease management and physical, psychological, spiritual, and social support to alleviate the suffering of patients and their caregivers and, thus, improve their quality of life. Healthcare professionals have generally agreed that PD is now a chronic, lifelong condition with no practical way to slow or stop the progression of the disease.

“I think PC is needed because we don’t have any particularly good way to get patients to eat and drink well, relieve their symptoms, alleviate some pain, extend the length of life appropriately and give them more dignity.” [Health professional].

##### **The desire for PC knowledge among health professionals**

Health professionals mentioned the absence of systematic education and training concerning PC services and expressed their eagerness to learn about the services to help improve their patients’ lives. They have also suggested that lectures and studies on PC be implemented at the hospital and set up relevant committees to oversee the effectiveness of the learning ecosystem.

“There is a great need for us to learn more about PC because if we know about PC, we can intervene the patients in the early stage of PD, and if patients are willing to know, we can introduce it to them.” [Health professional].

##### **Misconceptions about PC**

All populations have incorrectly perceived PC, including health professionals, patients, and caregivers. Two health professionals believed that PC does not include surgical treatment, i.e., a conservative treatment. One healthcare professional believed that PC does involve active intervention with patient’s treatments, while another thought that PC is similar to hospice care.

“I think the tendency is to be proactive, but not very proactive, a model of patient care for health care professionals that doesn’t actively force intervention in patient care …… Our initiative is not that strong.” [Health professional].

“I think it’s just conservative treatment because there’s nothing particularly good that can be done for this disease either, you can only do some management for the symptoms, it’s all up to the patient to take the medication on their own initiative and survive with the disease …….” [Healthcare professional].

##### **Economic burden**

Most PD patients mentioned the financial burden associated with the need for expensive medications used in the treatment. The concern was validated by health professionals, mentioning that PD patients usually need long-term medication, which is not yet covered by medical or health insurance.

“In the middle and late stages, I have to take a huge number of drugs, some of which are imported and particularly expensive, so it’s a little difficult to take the medication and lay a burden on my child.” [Patient].

“Now only Levodopa is covered by health insurance, others are not, so patients who can’t afford these drugs may not take them, so they can’t get relief from their symptoms.” [Policy maker].

#### Interpersonal level

At the interpersonal level, the central theme facilitating PC is social support. In comparison, lack of communication time and lack of multidisciplinary collaboration were identified as barriers to providing PC for patients with PD.

##### **Social support**

Health professionals expressed the need for social support among PD patients as they are prone to anxiety and depression following the diagnosis. Examples include the yearning for attention from medical staff among hospitalized patients. The introduction of PC that involves the roles of team members from multidisciplinary is believed to provide the care and support needed by PD patients.

“Patients just want to have us talk to them more…… With PC, the involvement of more team members can increase this support for patients and carers.” [Healthcare professional].

##### **Lack of communication time**

Most health professionals mentioned their busy work schedules with no time to spare for communicating with patients or providing knowledge to them beyond essential services. However, two health professionals said the need for nurses to be patient and regularly educate the patients.

“Usually, we undertake the basic life care of patients and occasionally carry out psychological care, but we basically don’t have much time. We can’t finish our own work at ordinary times, and we don’t have extra time to communicate with patients about these things (refer to PC).” [Health professionals].

##### **Lack of multidisciplinary collaboration**

Most health professionals indicated that the administration of Chinese medicine and psychological assistance are also needed in addition to medical treatment and rehabilitation. However, the cooperation between the various disciplines was not particularly close, and that multidisciplinary joint management was only carried out after the appearance of the appropriate symptoms. One healthcare professional even indicated that there is no need for multidisciplinary collaboration.

“As far as the hospital is concerned, I think there needs to be more of this multidisciplinary cooperation and closer collaboration.” [Health professional].

“I don’t think interdisciplinary cooperation is very good at the moment, only when patients present with particularly obvious symptoms, such as psychological reasons or other, do they actively seek multidisciplinary cooperation, and this is not particularly well connected to ensure easy interdisciplinary communication throughout the patient’s stay.” [Health professional].

#### Organizational level

At the organizational level, two main themes facilitating PC were identified: Investments towards systematization of PC and nurses are the bridge between patients and doctors. Meanwhile, the lack of PC specialist nurses and criteria for PC and the absence of economic benefits indicators were identified as barriers to providing PC for patients with PD.

##### **Investments towards systematization of PC**

According to several health professionals, hospital administrators are paying more attention to emerging PD patients. They are ready to form specialized groups and conduct relevant knowledge lectures, signifying a positive future for PC services. Two policymakers mentioned that the state is currently boosting its investment in PD. With the increase in PD patients, the state is taking various measures to improve the quality of life of PD patients.

“Our hospital has provided us with lectures on PC and is also gradually establishing this Parkinson’s disease center or alliance to facilitate understanding of the significance of PC and provide a relevant platform.” [Health professional].

##### **Nurses are the bridge between patients and doctors**

Most health professionals believe that nurses are responsible for connecting patients with doctors with a growing nursing workforce and the addition of educated nursing personnel. Therefore, nurses must be able to observe patients’ symptoms and communicate with doctors, acting as a “sounding board” in the middle.

“The nurse can identify the patient’s symptoms, give feedback to the doctor, who gives appropriate treatment, and the nurse can do a comprehensive assessment to give feedback……. The nurse is the connecting point between the doctor and the patient, passing on information.” [Health professional].

##### **Lack of PC specialist nurses**

Several nurses mentioned the lack of understanding among nurses in China about PC and the appropriate PC specialist nurses.

“If this (PC) is to be developed in the future, specialist nurses will definitely have to be trained and such specialist clinics will have to be opened. Nowadays, in an aging society, young people are unable to take care of them, and there is no specialized institution to provide support in this area.” [Health professional].

##### **Lack of referral criteria for PC**

When it came to implementing PC, most health was divided. Six health professionals believed that PC should be implemented from the early stages after the diagnosis to improve the patient’s quality of life; four health professionals believed that PC should be implemented from the mid to late stages of the disease when symptoms are more pronounced; two health professionals believed that PC should be implemented throughout the disease term.

“I think it makes more sense in the early stages when in the late stages (patients) are on all kinds of medications with all kinds of complications. It makes more sense to me to intervene in the early stages (of the patient), and with the care of the family, the disease at least progresses more slowly, and in the late stages (of the disease), it may not make much sense.” [Healthcare professional].

“I would choose to implement PC in patients with advanced disease because it would be a psychological burden for the patient to talk about it at an early stage.” [Healthcare professional].

##### **Absence of economic benefits indicators**

Several health professionals stated that it would take time to observe the effect of PC in patients, which is further complicated by the lack of appropriate feedback mechanisms.

“Right now, PC is slow to see results, so leaders may not invest in this area, and there is a lack of funding to carry it out.” [Health professional].

#### Community level

At the community level, two main themes facilitating the implementation of PC are the convenience of community services and a hospital-community-family-based services. Meanwhile, lack of PC resource access and discontinuity of care were identified as barriers to PC for patients with PD.

##### **The convenience of community services**

Most patients stated that community centers are developing rapidly, making it easy to buy medicines and seek advice regarding their illnesses. These patients also eagerly welcome the introduction of PC in their treatment routines.

“Our community hospital is well-built, sometimes some minor illnesses, I usually go directly to the community to buy medicine. It is very close and particularly convenient. If this PC could be opened in the community hospital, it would be very good. I would definitely be willing to go.” [Patient].

##### **A hospital-community-family-based service**

Considering the hierarchical medical system implemented in the country, several healthcare professionals expressed the need for tertiary hospitals to take the traditional role of training doctors and nurses. This includes allowing health professionals from lower-level hospitals to participate in the training program at the tertiary hospital. It was also proposed that the tertiary hospital should drive the secondary and community hospitals, and families, which can then use their geographical advantages to reach thousands of households, making it easier, faster, and more direct to guide families in managing their illnesses. An ecosystem that can improve the quality of treatment provided to the community, including PC, can be created through this relationship structure.

“Tertiary hospitals develop treatment plans, which are then dovetailed by the community institution to which the patient belongs, and send basic information about the patient down to the community, where the community follows up accordingly according to the tertiary hospital’s plan and continues with other treatments, which also helps patients with PC.” [Health professional].

##### **Lack of PC resource access**

Most patients reported the unavailability of PC services in their community and the lack of relevant information. Healthcare professionals supported the statement by mentioning the lack of popularity among the population, with most people still unaware of it, and the unavailability of PC-related services in the community.

“I still hope that the community can provide such an environment so that we know about this (PC) …… right now, it’s mainly because we don’t know much about it (PC).” [Caregiver].

##### **Discontinuity of care**

According to some health professionals, there is currently a lack of continuity of treatment for PD patients, with many patients coming in for one appointment and not necessarily returning the next. Furthermore, the present pandemic makes it more difficult for patients to maintain continuity of treatment, making PC a challenge.

“At the moment, the state is reducing the number of days in hospital, and patients have to be discharged in a hurry; some of them start to move around after discharge, and we can’t offer them any PC, you may just talk to them about this today, and they will be discharged tomorrow.” [Health professional].

#### Culture and policy level

The central theme facilitating PC delivery is an existing policy at the cultural and policy level. Meanwhile, cultural and ethical dilemmas were identified as barriers to providing PC for patients with PD.

##### **Existing policy**

Several healthcare professionals mentioned that the state is now focusing on chronic diseases and already has relevant PD drugs covered by health insurance. Gradually, health insurance will cover more PD drugs to reduce the financial burden on patients and families.

“At present, the state is very concerned about these chronic diseases, like some PC policies for cancer are better, and some places can learn from them, that is, there are some universal PC policies.” [Policy maker].

##### **Culture of death**

According to most patients and family members, health professionals often avoid discussing death-related topics as death is taboo according to traditional Chinese culture. Also, patients in this country typically do not opt for advanced life support in the final stage of the disease to avoid this taboo.

“No way, I can’t be involved in such a discussion, as long as I don’t give up resuscitation until the last minute, and we don’t talk about death with patients.” [Caregiver].

“In China, it is unlikely to discuss this issue of life and death with patients, and many times when it comes up, caregivers are reluctant and change their face a little bit, and they are psychologically unable to bear it.” [Health professional].

##### **Ethical dilemmas**

As filial piety is vital in China, discussing life and death or signing an agreement to give up resuscitation or initiate PC is considered unfilial and faced with moral condemnation. Healthcare professionals often find themselves caught in an ethical dilemma without a decision-maker in the family to discuss the patient’s treatments.

“China advocates filial piety. If you are not filial, you will be accused by your neighbors. It is impossible to discuss advanced care planning. Your family members will not allow you to do so first.” [Health professional].

“China used to implement family planning. Many of them were one-child families. The children were often absent, just him and his wife and children. They lacked child care, and it was difficult to communicate with their children about PC.” [Health professional].

## Discussion

This study offered profound perceptions on how stakeholders see the provision of PC to PD patients. Investigating the factors impacting PC delivery among patients with PD is crucial and timely, given the growing concern about the quality of life for people with PD. We found interactions between the influencing factors functioning at various levels using the socio-ecological model, which filled a gap in exploring influencing factors in prior studies. The specifics are provided below.

At the individual level, we observed a strong desire to learn about PC among patients, caregivers, and health professionals. Patients and caregivers have expressed their readiness to undergo PC to relieve PD symptoms and enhance their quality of life. Meanwhile, health professionals are ready to learn more about PC to be PC educators. The previous study [25] identified the ten most distressing symptoms in people with end-stage PD, including pain, stiffness, swallowing/feeding difficulties, psychological problems, etc. The study also revealed that these patients were willing to receive PC services, which are currently unfulfilled. Another study [26] noted a high level of receptiveness to PC among carers. Notably, reports on factors influencing PC incorporation in PD treatment have focused primarily on Western cultures [27, 28]. It is generally known that patients in Chinese cultures may have more difficulty accepting PC than those in Western cultures due to traditional beliefs about life and death. However, our findings demonstrate a promising PC future in China: most patients and carers were willing to undertake PC. Similar to previous studies [29, 30], our results also demonstrate that PD health professionals are motivated to learn about PC. These findings suggest a platform for learning should be created, and access to internet resources should be provided for health professionals to reach out to caregivers in the area with economic disadvantages.

Additionally, the study identified “ Misconceptions about PC” and “economic burden” as barriers at the individual level. Misunderstood that PC is equal to giving up treatment is one of the common misconceptions as PC was initially provided for patients with advanced cancer and only recently introduced to neurology [31]. This misconception has prompted some national and international experts [32] to request the renaming of PC. Studies have also revealed [33, 34] that professionals and patients who have been directly educated about PC show positive attitudes towards PC, indicating the need to educate patients and families timely. Currently, PC services for patients with PD are not yet covered by health insurance in China and patients face a significant economic burden, which is a deterrent for many patients. However, as PC development becomes more apparent, health insurance is expected to expand coverage for PD.

At the interpersonal level, our findings revealed that several patients and caregivers expressed a need for social support, which can be fulfilled through the PC program. PC focuses on the physical, spiritual, social, and psychological aspects of care for the patient and their carers [10]. The involvement of team members across multiple disciplines can overcome the shortcomings related to the lack of social support for patients and carers [35]. Research has demonstrated the high emotional burn and the need for support among the carers for PD patients [36]. Similarly, our study revealed that the limited time for communication and lack of multidisciplinary collaboration are the barriers to providing PC for PD patients and their carers at the interpersonal level. Our findings also demonstrate that developing an excellent trusting relationship between doctor and patient is a crucial prerequisite for PC and that better communication can help with this. However, the current healthcare status in China is marked by a significant imbalance between the number of patients and doctors and nurses [37], restricting the time for good communication between doctors and patients and delaying PC implementation. Multi-disciplinary team is a patient-centered clinical discussion involving a multidisciplinary team (two or more specialists) working to develop a standardized, individual, and sequential approach for optimal treatment for a specific disease [35]. It is the high-quality multidisciplinary collaboration that forms the basis for PC. However, the uneven development of different clinical disciplines, especially in rehabilitation, makes developing PC for PD patients ineffective.

Concerning the organizational aspects of PC services for PD, we found that three critical barriers need to be addressed: the lack of PC specialist nurses, the lack of referral criteria for PC, and the absence of economic benefits indicators. Indeed, there is no specific timing regarding the initiation of PC in PD patients [38], which tends to hinder PC delivery to PD patients. Some scholars support the initiation of PC based on patient needs [17, 39, 40], whereas others support the initiation based on disease progression [41, 42]. Most studies have proposed that PC should be initiated in PD patients with Hoehn & Yahr (≥ 3 or ≥ 4) [43–46], with further detailed guidelines should be issued in the future to standardize the delivery of PC to PD patients. Our study also identified two organizational management factors that could facilitate the delivery of PC services. The first is the investment of policy leaders and hospital administrators toward developing PC services; the second is nurses are the bridge between patients and doctors who can fully dedicate themselves to providing a PC services. Considering the direct and frequent contact between nurses and patients, nurses may be the best people to communicate the patient’s needs. Most previous studies [47, 48] have shown that nurse-led PC services effectively improve patient and carer life quality, indicating the condition to fully utilize their roles to reduce healthcare costs and promote PC for PD patients.

Our results also revealed that at the community level, patients with PD have difficulty accessing services related to PC, and there is a lack of continuity of care for patients with PD. However, community health care services and a hospital-community-home service chain are more convenient for patients. Most patients in China seek care in general tertiary care hospitals, which can be very costly and burdensome. Therefore, the development of PC services in the community is welcomed. Patients have reported that if primary health care meets their needs, they are more inclined to visit a hospital “near their doorstep”.

At the level of culture and policy, this study identified two barriers and one facilitator. The two barriers include “culture of death” and “ethical dilemmas”, The absence of knowledge on life and death in China has left education in the area at a rudimentary level. Given the influence of traditional Chinese culture, Chinese people emphasize “birth” and avoid talking about “death”. This has led to a “fear of death” among the Chinese population. This cultural belief also makes patients with PD and their carers uncomfortable with PC. The cultural idea is considered a major barrier [49], indicating the need for healthcare professionals to consider different cultural beliefs when delivering PC interventions and choose a more acceptable approach for patients and carers. Previous studies have similarly found that [50, 51] healthcare professionals performing PC are vulnerable to ethical dilemmas. In the management of PD, health professionals are also faced with ethical dilemmas: “whether to suggest PC to patients”; “how to suggest PC to patients”; “what if patients cannot accept PC and it causes more conflict?”. These ethical dilemmas are more prominent in developing countries with a traditional background. In PC settings, ethical questions must be handled cautiously with high sensitivity, considering cultural traditions and budgetary restraints. Potential involvement in assisting decision-making in PC settings should be researched and debated. It is worth mentioning that China is developing PC and has introduced a series of policies in this regard.

### Strengths and limitations

To the best of our knowledge, this is the first study to use the social-ecological model to explore the factors influencing PC delivery to patients with PD from the perspectives of patients, carers, health professionals, and policymakers. Although topic saturation was attained, the number of participants in this study was small and confined to health professionals, patients, caregivers and policy makers living in the middle of China. More research is needed using the sample from the population of health professionals, patients, caregivers and policy makers varied by race and gender across the country. In Addition to this, results are inevitably influenced by researcher subjective bias and to minimize the impact of subjective bias on our research, we invite interviewees to review their interview texts, hold regular team meetings and invite sceptical peers to review data material.

### Implications for policy and practice

Our study uses an ecosystemic theoretical framework to identify the factors influencing PC delivery to patients with PD at five levels. The findings help healthcare professionals, managers, and policymakers resolve problems at their level and improve PC for patients with PD.

First, PD patients, carers, and health professionals should receive more PC education. Adopting interventions like the Knowledge-Attitude-Practice model may dispel misconceptions about PC and increase acceptance of it. Online platforms can also facilitate the dissemination of PC messages during the epidemic.

Second, the system for providing ongoing care to PD patients should be enhanced, including creating a library of ethical cases concerning PC delivery to help health professionals handle any ethical dilemmas that could arise.

Third, to make PC more accessible to patients and caregivers, policymakers in the domain of PD or PC should be urged to integrate PC services in health care coverage gradually.

Fourth, it is crucial for PC experts in the domain of PD to promptly clarify any critical issues, such as the referral time and prognostic indicators, to facilitate the development and improvement of guidelines. Doing so will help health professionals deliver high-quality PC services for patients and caregivers.

ions, expert testimony, grants or patents received or pending, or royalties. Peer reviewers on this manuscript have no relevant financial or other relationships to disclose.

## Electronic supplementary material

Below is the link to the electronic supplementary material.

Supplementary Material 1

## Data Availability

The data set generated and analysed during the current study will not be shared to maintain participants’ anonymity and confidentiality but are available from the corresponding author on reasonable request.

## Abbreviations

PC: Palliative care
PD: Parkinson’s disease
SEM: socio-ecological model
WHO: The World Health Organization
