# Supplementary material for: Facilitators and barriers to the delivery of palliative care to patients with Parkinson’s disease: a qualitative study of the perceptions and experiences of stakeholders using the socio-ecological model
Source: BMC Health Serv Res. 2023 Mar 6;23:215. doi: 10.1186/s12913-023-09203-2 (PMC9990289; doi:10.1186/s12913-023-09203-2)
Supplement: Supplementary file 1 — Supplementary Material 1 [file 12913_2023_9203_MOESM1_ESM.docx]

**Supplementary file 1**

Interview guides

Interview for health professionals

| Health professionals |
| --- |
| Have you ever been in contact with someone with Parkinson’s disease? How many connections can you have in a month?  What are the general symptoms of patients with different stages of Parkinson’s disease? How do you manage and relieve the symptoms of these patients?  Have you ever heard of the term palliative care/palliative medicine? What is your understanding of this term?  Have you ever heard of palliative care for Parkinson’s disease patients?  -Yes, can you tell me exactly how it is done?  -No. Do you think palliative care is necessary for people with Parkinson’s disease? What is the most appropriate stage of the disease to start?)  If palliative care for patients with Parkinson’s disease were to be carried out, which healthcare professionals do you think would be needed and what would be the division of labor among them? What role could you take in the palliative care of patients with Parkinson’s disease?  Do you think it is necessary for people with Parkinson’s disease to know about palliative care in the early stages of the disease?  Do you think health professionals with Parkinson’s disease must learn about palliative care?  Do you know places currently offering palliative care services for people with Parkinson’s disease? And what do you think are the recommended places where palliative care for people with Parkinson’s disease could be carried out in the future?  Do you think Chinese culture has an impact on palliative care for Parkinson’s disease patients? Where exactly? Can you give me an example?  How do you think hospital leaders, community or national health policies, etc., can promote this palliative care for people with Parkinson’s disease? |

Interview for patients with Parkinson’s disease

| Patients with Parkinson’s disease |
| --- |
| How long have you had Parkinson’s disease?  Which symptoms of Parkinson’s disease do you think affect you more? What are the symptoms that are more difficult for you to deal with?  What methods do you currently use (have you ever used them) to deal with these symptoms?  How do you usually find out about Parkinson’s disease and your prognosis?  How would you describe your social and interpersonal life after being diagnosed with Parkinson’s? Has it changed at all?  What do you feel is your biggest motivation to stay with your treatment?  Have you heard of the term palliative care/palliative medicine?  --Yes, what is your understanding of it?  --Never heard of it? Give a brief introduction  As a person with Parkinson’s disease, would you undergo palliative care? Why?  What do you think of the end of your life (death)?  What support do you currently receive? (from the state, community, hospital, and family)  What other support and help would you need to undertake palliative care in the future? |

Interview for caregivers

| Caregivers |
| --- |
| What is your relationship with the patient? How long have you been caring for them?  What symptoms do they usually have? What do you do to alleviate and care for their symptoms?  In what ways do you learn about Parkinson’s disease and the patient’s prognosis?  Have you heard of the term palliative care/palliative medicine?  --Yes, what is your understanding of it?  --Never heard of it? Give a brief introduction  As a carer, would you support a patient in going for palliative care? Why?  What support do you currently receive? (from the state, community, hospital, and family)  What other support would you need if you were to start palliative care for people with Parkinson’s disease in the future?  As a carer, what factors do you feel would prevent you from participating in palliative care?  As a carer, what do you see as the future of the disease? Have you ever talked to a patient about ‘death’? What does the person want to do if they cannot speak for themselves one day?  ---If so, what can you tell me about that conversation?  ---If not, when do you think it would be appropriate to start such a conversation? |

Interview for policymakers

| Policymakers |
| --- |
| Have you heard of palliative care/palliative medicine?  What do you think about palliative care for people with Parkinson’s disease?  Are there any policies in place for palliative care for people with Parkinson’s disease?  --Yes, can you give me a list?  --No. Do you think there is a need for such a policy? Why?  What do you think are the barriers and facilitators to implementing policies on palliative care for people with Parkinson’s disease? |

**Supplementary file 2**

Socio-ecological model

Ecology is the study of the interactions between biological communities and their environments[1]. It has its roots in the biological sciences. Social ecology is the study of interactions between people and the environment, primarily at the human-social-natural level (also known as ecosystem theory in sociology and social work). It is an investigation of the human, social, and ecological spheres[2-3]. According to social ecology, rather than viewing individual behavior as the consequence of the individual alone, it is the outcome of the interaction between the individual and the environment. The physical and social environments make up the majority of the environment. The physical environment and the social environment are the two fundamental components of the environment[4].

Reference:

1. Bronfenbrenner U, Ceci SJ. Nature-nurture reconceptualized in developmental perspective: a bioecological model. Psychol Rev 1994; 101(4):568-86.

2. Jones T, Luth EA, Lin SY, Brody AA. Advance Care Planning, Palliative Care, and End-of-life Care Interventions for Racial and Ethnic Underrepresented Groups: A Systematic Review. J Pain Symptom Manage 2021; 62(3):e248-e60.

3. Harden KP, Turkheimer E, Loehlin JC. Genotype by environment interaction in adolescents' cognitive aptitude. Behav Genet 2007; 37(2):273-83.

4. Lee BC, Bendixsen C, Liebman AK, Gallagher SS. Using the Socio-Ecological Model to Frame Agricultural Safety and Health Interventions. J Agromedicine 2017; 22(4):298-303.


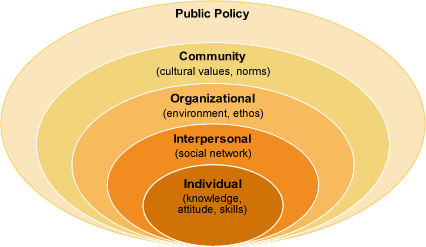


Figure S1: Social-ecological model
